# Taxonomic revision of *Mussaenda wallichii* (Rubiaceae): two new synonyms from Southeast Asia

**DOI:** 10.3897/phytokeys.280.209108

**Published:** 2026-10-14

**Authors:** Ting-Ting Duan, Yi-Xuan Li, Jin Li

**Affiliations:** 1 Guangdong Ocean University, Zhanjiang CN-524088, Guangdong, China Guangdong Ocean University Zhanjiang China

**Keywords:** *
Mussaenda
*, nomenclature, Rubiaceae, synonymy, taxonomy

## Abstract

*Mussaenda wallichii* G. Don (Rubiaceae) is a widespread species distributed from northeastern India to Southeast Asia, but its taxonomic delimitation has remained uncertain due to overlapping morphological characters and incomplete assessment of type materials. Based on extensive herbarium examinations, including type specimens, and critical evaluation of protologues, we reassessed the taxonomic identities of *M. intuspilosa* Jayaw. and *M. lancipetala* X.F. Deng & D.X. Zhang. Morphological comparisons revealed that both taxa are conspecific with *M. wallichii* and cannot be distinguished from it by consistent diagnostic characters. Therefore, they are treated as heterotypic synonyms of *M. wallichii*. Lectotypification of historical names is also discussed. This study furthers our understanding of morphological variation, species diversity, and distribution patterns within *Mussaenda* in Southeast Asia.

## Introduction

*Mussaenda* L. (Rubiaceae) is a paleotropical genus comprising approximately 135 species distributed mainly in tropical Africa and Asia (Alejandro et al. 2005). The genus is considered to have originated in Africa and subsequently diversified throughout tropical Asia (Alejandro et al. 2005; Duan et al. 2018). More than 50 species are currently recognized in the Indochina Peninsula, which is considered as one of the major diversity centers of the genus (Hooker 1880; Gagnepain 1923; Jayaweera 1963; Chen and Taylor 2011; Chantaranothai 2015; Duan et al. 2018). Although some taxonomic studies have mainly focused on several discrete regions including China, India and Thailand, an integrated taxonomic revision is still expected (Jayaweera 1963; Deng 2007; Chen and Taylor 2011; Chantaranothai 2015; Duan et al. 2016, 2019; Huang et al. 2025). The current classification of *Mussaenda* is mainly based on morphological characters, including leaf morphological (shape, venation, indumentum), stipule morphology, calycophyll morphology, relative lengths of calyx tubes versus lobes, and the shape of calyx and corolla lobes. (Deng 2007; Chen and Taylor 2011; Zhang et al. 2024).

**Figure 1.**
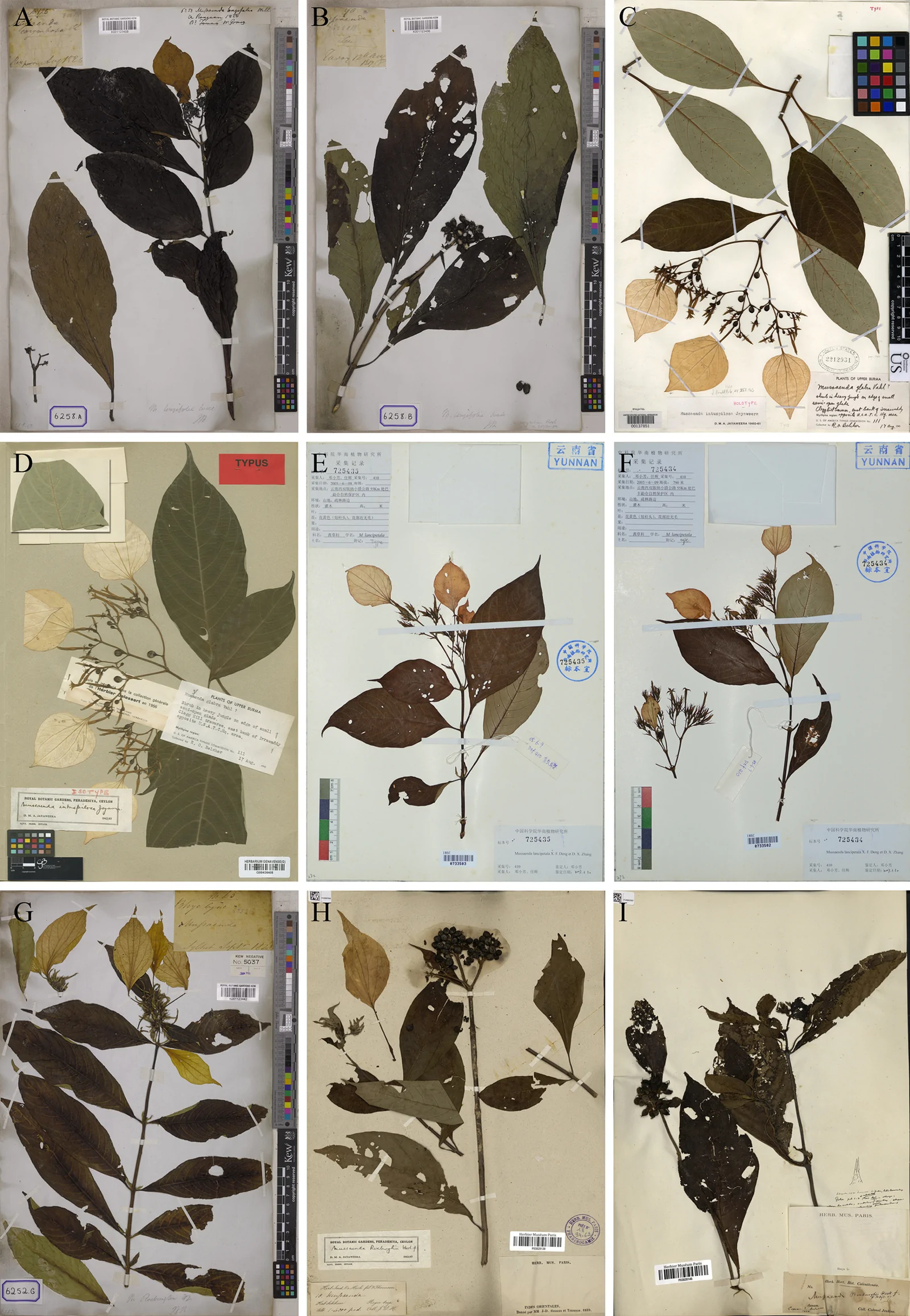
Herbarium specimens of *Mussaenda wallichii* G. Don and related taxa. **A**. Lectotype of *M. wallichii* (K001123455); **B**. One sheet of the original material of *M. wallichii* belongs to *M. roxburghii*. **C, D**. Holotype and isotype of *M. intuspilosa* Jayaw. **E, F**. Lectotype and isolectotype of *M. lancipetala***G–I**. Holotype, paratypes of *M. roxburghii*.

*Mussaenda wallichii* G. Don was published in 1834, with a brief protologue and voucher specimens collected from “Tavoy and Rangoon” in southern Myanmar and labelled “Wall. cat. no. 6258” (Fig. 1A, B). Meanwhile, Don (1834) clearly pointed out that these two voucher specimens did not represent *M. longifolia* Lam. (Lamarck & Poiret, 1797), as Wallich had annotated on the specimen labels. Our examination of these two type materials showed that the two specimens which are labelled Wallich 6258A and 6258B, respectively, are morphologically distinct. Specimen 6258A exhibits morphological characters consistent with the protologue description of a terminal, trichotomous inflorescence. In contrast, specimen 6258B, a fruiting branch specimen, bears densely congested, subcapitate berries with short pedicels, which do not match the diagnostic traits of this species. After extensive comparisons with numerous specimens and relevant literature, we think that the morphological characters of 6258B match those of *M. roxburghii* (Fig. 1G–I). The primary distinction between the two taxa lies in their inflorescence morphology: the former has spreading corymbs, whereas the latter possesses capitate cymes. Detailed comparisons between these two species are listed in Table 1. Because the previous lectotypification of Wallich 6258 did not specify a single specimen among the original materials, and one of the specimens (6258B) is referable to *M. roxburghii*, we designate Wallich 6258A as the second-step lectotype in accordance with ICN Art. 9.17.

**Table 1.** Morphological comparison of *Mussaenda wallichii*, *M. intuspilosa*, *M. lancipetala* and *M. roxburghii* based on type specimens, protologues, and examined specimens.

**Characters**	***M. wallichii***	***M. intuspilosa***	***M. lancipetala***	***M. roxburghii***
Stem	Shrub	Shrub	Shrub	Shrub
Leaves	Long elliptic, glabrous	Oblong-lanceolate or elliptic-lanceolate, 8.5–24 × 3–8 cm; glabrous adaxially, minutely puberulous abaxially	Oblong or elliptic, 8–24 × 3.5–8 cm, glabrous adaxially, sparsely pubescent abaxially	Oblong-lanceolate, ovate or elliptic, 9–28 × 3.5–9.8 cm
Secondary veins	8–11 pairs	8–10 pairs	8–11 pairs	7–14 pairs
Petiole	0.6–3 cm glabrous	0.5–3 cm long, glabrous or puberulous	0.5–3.0 cm long, sparsely pubescent	0.5–2.5 cm long, glabrous or puberulous
Stipule	Triangular-lanceolate,	Triangular-lanceolate,	Triangular-lanceolate,	Triangular-lanceolate
Inflorescence	Terminal, trichotomous	Terminal, trichotomous	Terminal pleiochasium;	Terminal, capitate cyme
Bracts & bracteoles	Lanceolate	Lanceolate	Lanceolate,	Bracts lanceolate; bracteoles trilaciniate into short or long linear segments
Calyx lobes	Lanceolate	Lanceolate,	Lanceolate	Lanceolate
Petaloid sepal	Ovate or elliptic-lanceolate, glabrous to minutely puberulous	Ovate or elliptic-lanceolate, glabrous to minutely puberulous	Ovoid, glabrous to minutely puberulous	Oblong-lanceolate, pubescent
Corolla tube	2.5–3.0 cm long, glabrous or minutely pubescent outside	2.5–3.0 cm long, glabrous or minutely pubescent outside	2.5–3.0 cm long, sparsely pallid farinose outside	2.4–2.8 cm long, appressed-pubescent outside
Corolla lobes	Lanceolate	Lanceolate	Ovate-lanceolate	Oblong-lanceolate,
Fruits	Elliptic to ovoid, with deciduous calyx lobes	Elliptic to ovoid, with deciduous calyx lobes	Elliptic to ovoid, with deciduous calyx lobes	Globular, with persistent calyx lobes

*Mussaenda intuspilosa* Jayaw. was published with a complete series of specimens collected from Assam (India), Upper Myanmar and Lower Myanmar, with the collection *R.O.Belcher* U. S. A. Typhus Comm. 111 (US) as the holotype (Fig. 1C, D)(Jayaweera 1963). In the protologue, the author noted that this taxon was once tentatively assigned to *M. glabra*, from which it can be clearly distinguished by its larger leaves, larger stipules glabrous or pubescent along the midline and base on the outer surface, pubescent within at the base with numerous glands, sepals larger. It is easily separated from *M. roxburghii* by its diffuse inflorescence, lanceolate sepals glabrous or minutely pubescent outside the corolla tube, and the calyx segments deciduous from the fruit. In addition, the author also observed intraspecific morphological variation across populations, including differences in indumentum density, corolla tube length, and the shape of glands at the stipule base.

In 2008, Deng and Zhang published *Mussaenda lancipetala* X.F.Deng & D.X.Zhang, a new species collected from Mengla, Xishuangbanna in western Yunnan, China (Deng and Zhang 2008). Although the inflorescence was described as a pleiochasial cyme, in most specimens it is trichasial, and tetrachasial cymes occur only occasionally. In addition, observations on numerous specimens and field study revealed considerable variation in the indumentum on the abaxial leaf surface and calycophyll. Individuals from some populations possess nearly glabrous leaf blades and calycophylls, with pubescence confined primarily to the midribs, whereas others are distinctly pubescent. This character shows no correlation with geographic origin. Such variation may be associated with different habitats or ontogenetic stages. The original authors noted this taxon is closely allied to *M. roxburghii* Hook.f., yet distinguishable by its larger laminar size, entirely glabrous adaxial leaf surfaces, more numerous pairs of secondary veins, diffuse inflorescences, and longer corolla tubes and lobes. The authors simultaneously cited the same gathering *X.F. Deng & H. Ren 410* (IBSC) as both holotype and isotype. The simultaneous designation of the same gathering as both holotype and isotype does not satisfy the requirements for designation of a single holotype, and the duplicate specimens should therefore be treated as original material requiring lectotypification. Under the International Code of Nomenclature for algae, fungi, and plants (ICN), the two duplicate sheets of *Deng & Ren 410* deposited at IBSC should be treated as syntypes (Turland et al. 2018).

**Figure 2.**
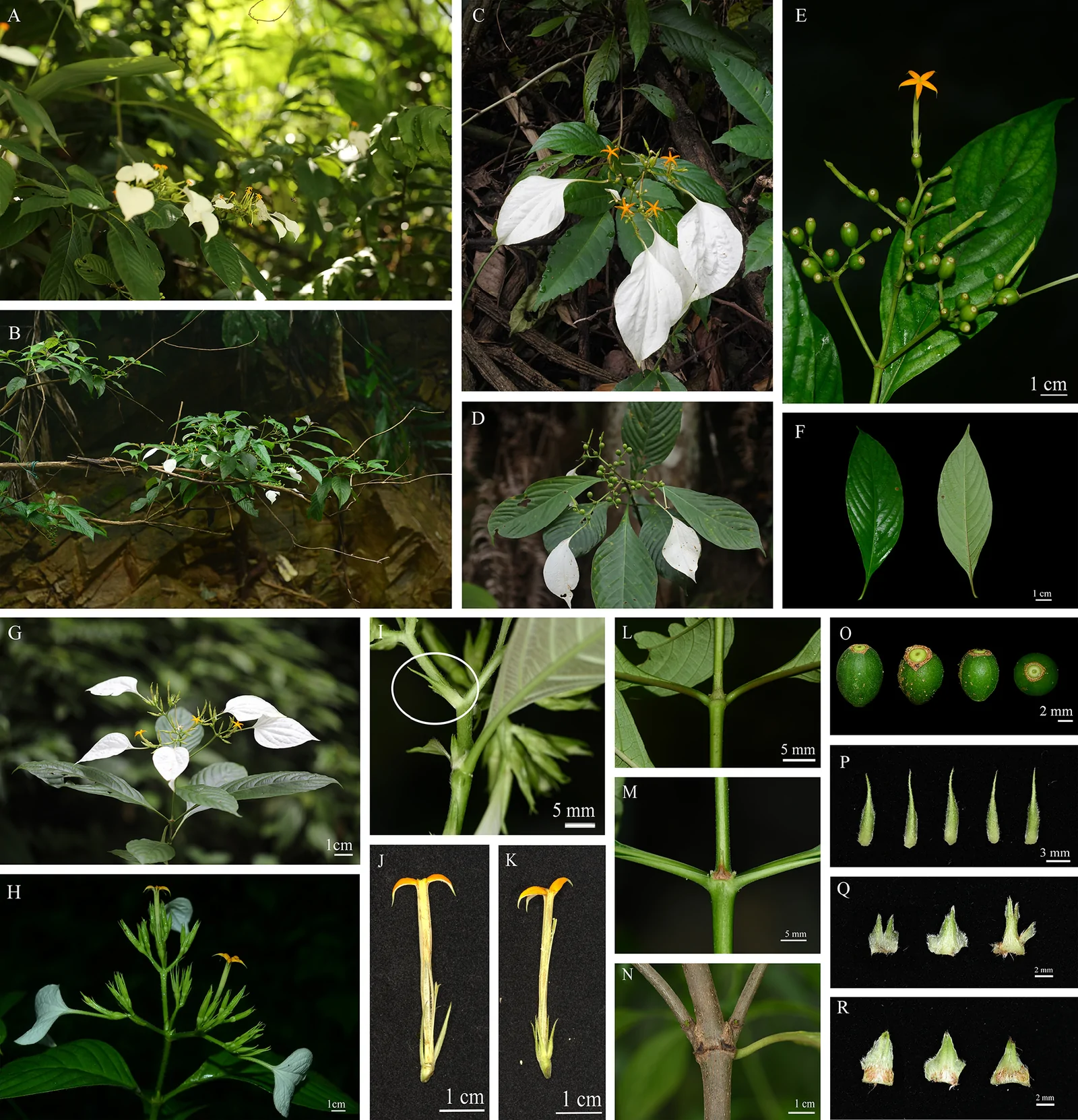
*Mussaenda wallichii* G. Don. **A, B**. Habitats and habit; **C**. Frontal view of infructescence; **D**. Front view of fruiting branch; **E**. Lateral view of fruiting branch; **F**. Leaves; **G, H**. Lateral view of infructescence; **I**. Bract; **J**. Longitudinally dissected, short-styled flower; **K**. Longitudinally opened, long-styled flower; **L, M**. Young branches; **N**. Old branch; **O**. Fruits; **P**. Calyx lobes; **Q**. Outer surface of stipules; **R**. Inner surface of stipules.

Comparative analysis of specimens (especially type specimens) and protologues of *Mussaenda intuspilosa*, *M. lancipetala* and *M. wallichii* (Table 1), as well as field observations of living plants during our taxonomic study of *Mussaenda* in Indochina Peninsula (Fig. 2), demonstrated that they are conspecific and represent the same species, and no stable morphological characters were found to distinguish them, such as oblong-elliptic leaves, cuneate at base and acuminate at apex, terminal trichotomous cymes, ovoid to elliptic berries. Notable differences among the three protologues include: *M. wallichii* was described as entirely glabrous, while *M. intuspilosa* possesses glabrous stems, and *M. lancipetala* bears sparsely pubescent young branches that become glabrescent at maturity; in terms of leaf morphology and trichome distribution, leaves of *M. wallichii* are entirely glabrous, elliptic, those of *M. intuspilosa* are oblongo-lanceolate with only minute puberulence restricted to the abaxial surface, 8–10 pairs of lateral vein, and *M. lancipetala* has oblong-elliptic leaves with sparse adaxial hairs and dense pubescence along the abaxial midrib, secondary veins 11–13 pairs; additionally, the white calycine leaflets (semaphylls) of *M. lancipetala* are densely pubescent along the veins, whereas those of the other two taxa are glabrous. Our observations confirmed that young branches’ leaves of *M. wallichii* and *M. intuspilosa* also have as sparse pubescence as *M. lancipetala*. In addition, the calycine leaflets of the latter two taxa are also pubescent along the veins, as in *M. lancipetala*. We re-examined the type specimen of *M. lancipetala*, and its secondary veins are 8–11 pairs consistent with *M. intuspilosa* and *M. wallichii*, rather than the 11–13 pairs recorded in the original description; this discrepancy may result from the inclusion of secondary minor veins in the original count. In the protologue of *M. lancipetala*, its corolla lobes are described as caudate at the apex, whereas those of *M. intuspilosa* are lanceolate with merely an acuminate tip. Despite the distinct descriptions in their protologues, extensive observation of abundant specimens indicates that corolla lobes are caudate to acuminate at the apex, with variation in the length of the terminal tail. Other characters, such as stipule morphology and indumentum, petiole length, calycophyll size and calyx lobe morphology, and fruit morphology show negligible variation. Nearly all morphological traits exhibit extensive overlap among the three taxa, supporting their taxonomic unification.

The geographical distribution ranges of the three taxa are partially overlapped or continuous. According to the research of Chantaranothai (2015), *Mussaenda wallichii* is not only distributed in southern and southeastern Myanmar, but also occurs in the adjacent regions including India (Nicobar Islands), Thailand and Peninsular Malaysia. *M. intuspilosa* is distributed in Myanmar and the adjacent Assam region of India, whereas *M. lancipetala* occurs in southern and southwestern Yunnan, China. It can thus be seen that the distribution ranges of the three taxa form a continuous belt stretching from Assam of India, through the whole territory of Myanmar, to southern and southwestern Yunnan of China.

In this study, we aim to clarify the taxonomic status of *Mussaenda wallichii* and its related taxa through examination of type specimens, protologues, and extensive herbarium collections. Based on morphological comparisons and nomenclatural analyses, we demonstrate that *M. intuspilosa* and *M. lancipetala* represent heterotypic synonyms of *M. wallichii*. The results provide a more stable taxonomic framework for understanding species diversity within *Mussaenda*.

## Taxonomic treatment

### Mussaenda wallichii

Taxon classificationPlantaeGentianalesRubiaceae

G. Don, Gen. Syst. 3: 490. 1834; Mussaenda glabra var. wallichii (G. Don) Kurz, J. Asiat. Soc. Bengal, Pt.2, Nat. Hist. 46: 159 (1877).

7F746971-448F-572F-8EED-99BF2020A09A

Figs 1, 2, 3

= Mussaenda jelinekii Kurz, J. Asiat. Soc. Bengal, Pt. 2, Nat. Hist. 45(2): 135. 1876. Type: India, Nicobar Islands, Great Nicobar, *A. Jelinek 250 Expedition Novara 90* (W0068113).
= Mussaenda oblonga King, J. Asiat. Soc. Bengal Pt. 2, Nat. Hist.72(4): 186. 1903. Type. MALAYSIA. Perak, Larut, June 1881, King’s Collector 1935 (lectotype: K000740937, designated by Chantaranothai, Thai Forest Bull., Bot. 43: 63. 2016).
=Mussaenda intuspilosa Jayaw., J. Arnold Arbor. 44: 257. 1963, syn. nov. Type. MYANMAR. Kachin, Myitkyina region, Clegg Hill traverse, east bank of Irrawaddy, 17 August 1945, *R.O. Belcher* U. S. A. Typhus Comm.*111* (holotype: US 2212931; isotype: G00436408).
= Mussaenda lancipetala X.F. Deng & D.X. Zhang, J. Syst. Evol., 46: 220. 2008, syn. nov.

#### Type.

Myanmar. *Wallich 6258* (lectotype designated by Chantaranothai (2015): K-W; second-step lectotype designated here under ICN Art. 9.17: *Wallich 6258A*, K001123455).

**Figure 3.**
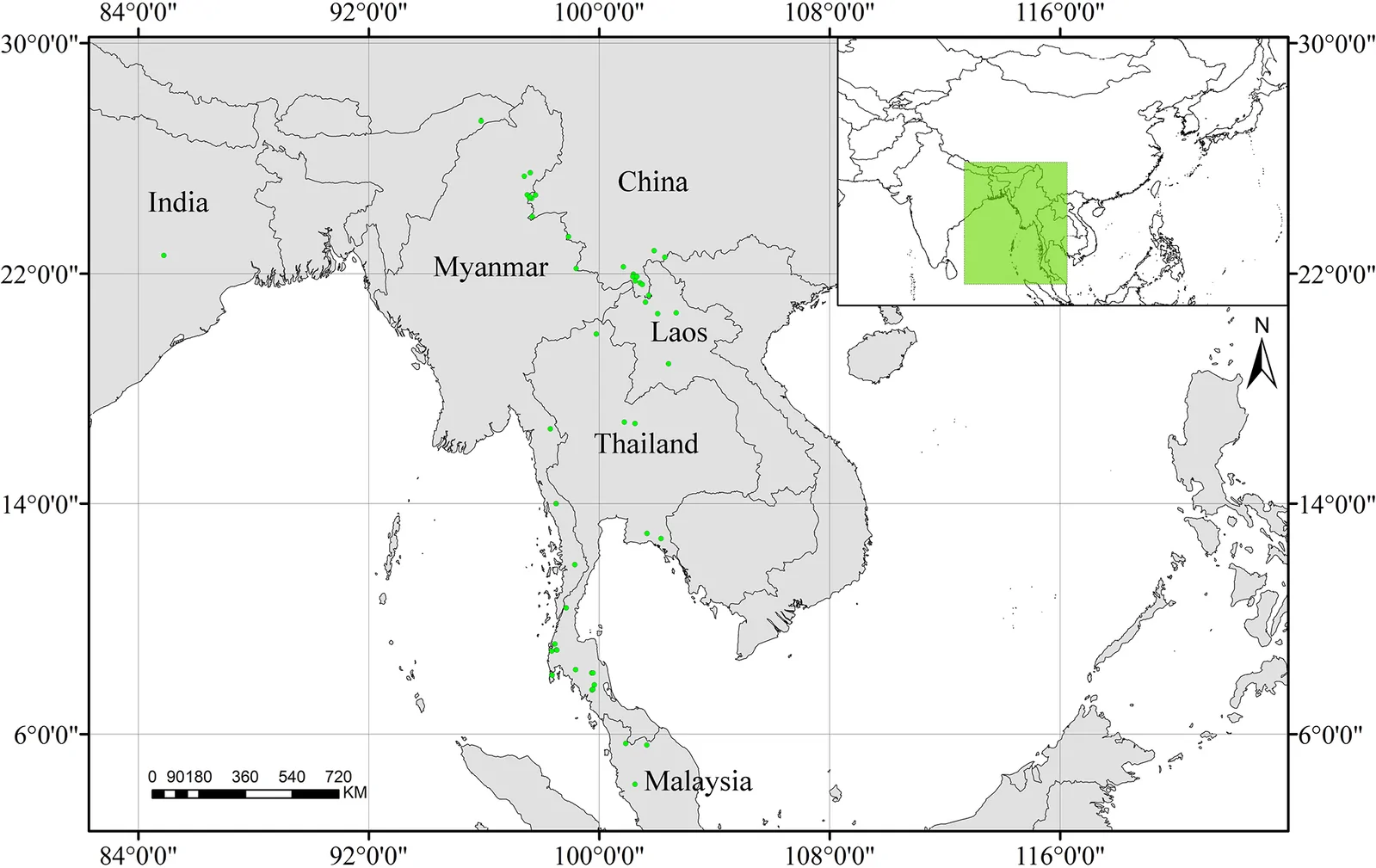
Distribution of *Mussaenda wallichii* G. Don.

***Lectotype*** (designated here). CHINA. Yunnan, Xishuangbanna, Mengla County, Menglun Town, in forest, 790 m, 9 June 2005, *X.F. Deng & H. Ren 410* (lectotype: IBSC0733503; isolectotype: IBSC0733502).

#### Description.

Shrubs, young branches angular, sparsely pubescent, becoming glabrescent when mature. Leaves oblong-lanceolate to elliptic-lanceolate, 8.5–24 cm long, 3–9 cm broad, acuminate, cuneate at base, glabrous on the upper surface, minutely puberulous beneath with 8–11 pairs of lateral veins prominent on the lower surface; petiole 0.5–3 cm long, glabrous or puberulous; stipules triangular-lanceolate from a broad base, 4.0–8.5 mm long, 3.0–6 mm broad at the base, bifurcate at the apex, pubescent on the outer surface, pubescent inside at the base and among numerous glands. Inflorescence terminal, usually trichotomous cyme, glabrous or minutely appressed-pubescent; bracts trifid or bifid; bracteoles lanceolate, 4–7 mm long, pubescent. Flowers heterostylous; pedicels stout, 1–2 mm long, glabrous or appressed-pubescent. Calyx tube 3 × 1.5 mm, elliptic-turbinate, sparse pubescent; calyx lobes five, lanceolate, 4.5–12 mm long, 1–1.7 mm broad, acuminate, pubescent on the outer surface; semaphylls white, ovate or elliptic-lanceolate, 4.5–10 cm long, 4.5–6 cm broad, short acuminate or acute, cuneate, glabrous on the upper surface, minutely puberulous beneath, longitudinal veins 5, “petiole” 1.2–3.0 cm long, minutely puberulous. Corolla tube 2.5–3.6 cm long, glabrous or minutely pubescent outside, densely yellow clavillose villous at amplification, thinly yellow pubescent below, corolla lobes 5–8 mm long, 2–2.5 mm broad, acuminate to caudate, glabrous on the outer surface and minutely papillate within. Stamens five, with short filaments, filament glabrous, adnate to the corolla tube below the middle, 2/5 way up in long-styled forms and 3/5 way up in short-styled forms; anthers linear, dorsifixed, introrse, 5–6 mm long, acute, bilobed at base. Ovary inferior, turbinate or globular, 3–4 mm long, minutely and scantily appressed pubescent; style and stigma lobes 2–2.7 cm and 2–2.5 mm long respectively in long-styled forms, 1–1.5 cm and 3–3.5 mm long in short-styled forms. Berry elliptic to ovoid, 6–9 mm long, 5–6 mm in diameter, glabrous or pubescent, with a deciduous calyx.

#### Chinese name.

狭瓣玉叶金花 (xiá bàn yù yè jīn huā).

#### Distribution.

India (Nicobar Islands and Assam), China (western and southern Yunnan), Myanmar, Thailand, Laos, and Peninsular Malaysia. (Fig. 3).

#### Habitat and ecology.

This species is found in a wide variety of habitats, including tropical lowland evergreen rain forests, dry evergreen forests, coastal dry evergreen forests, mixed forests on limestone hills, lower montane rain forests, and secondary forests. It is often found along roadsides, forest edges or stream valleys, along streams and in forest margins, from near sea level to approximately 1,000 m elevation.

#### Phenology.

Flowering from April to October, fruiting from June to December.

#### Conservation status.

*Mussaenda wallichii* G. Don is widely distributed from northeastern India to Southeast Asia, including China, Myanmar, Thailand, Laos and Peninsular Malaysia. Numerous populations have been recorded across its distribution range, and no evidence currently suggests significant population decline or severe habitat restriction. Following the IUCN Red List Categories and Criteria (IUCN Standards and Petitions Committee 2024), we assess *M. wallichii* as Least Concern (LC).

#### Specimens examined.

**India**. • Great Nicobar Island (Nevydera and Tendil forest area), 21 May 2003, *V.B. Shimvale 610* (K005313550; K005313551); • Nicobar Islands, 23 February 1858, *A. Jelinek 250* (W0068113); Assam, Namchik River Valley; 23-mile mark on Ledo Road, *R. O. BELCHER & G. E. JUAN*, U. S. A. Typhus Comm. *953* (US02371483); **Myanmar**. • Tanintharyi Region (Tenasserim Division), Dawei District (Tavoy): 1/2 mile east of Paungdaw Power Station, riverside, September 1961, *J. Keenan, U.Tun Aung & R. H. Rule 1466* (K005313556); • Tanintharyi Region (Tenasserim Division): Thebyu, Theinkum chaung, streamside, 08 June 1926, L. *T. Maung & C. E. Parkinson 1384* (K005313552; K005313554); Rangoon, s.d., *J. McClelland sn*. (K005313555); • Kachin, Myitkyina, east bank of Irrawaddy, Clegg Hiel traverse, edge of semi-open glade in dense jungle, 17 August 1945, *R. O. Belcher* U. S. A. Typhus Comm. *111* (US00137851; US00703127; G00436408); • Kawthule, Myawaddi to Kawkereik Hills, 21–23 December 1920, *J. F. Rock 712* (US00703126); ibid., 21–23 December 1920, *J. F. Rock 714* (US00703125); **China**. • Yunnan. Lincang City, Cangyuan County, Banlao Township, Huilun River, 31 October 1989, *G. D. Tao & X. W. Li 40050* (KUN 037873); • Ruili City, Yuhu Township, trail from Banling Port to Mengdian, 12 July 2013, *H.J. Dong, Z.H. Wang, G.X. Hu & Y.P. Chen RL-Z-042* (KUN1020867; KUN102088); • Pu’er City, Jiangcheng County, Tuka River, 28 August 1989, *Y. Y. Qian s.n*. (HITBC110058); Pu’er City, Menglian County, Mengma Township, Meng’a, mountain valley forest, 10 August 1973, *Menglian Survey Team 010051* (HITBC036713). Xishuangbanna, Mengla County, Yao District, Nazuo, 23 November 1974, *Z. H. Yang 012440* (KUN036675); • Xishuangbanna, Mengla County, ditch at 55 km on Xia-La Highway, 6 June 2014, *L.Wu 4795* (BNU0031300); • Xishuangbanna, Jinghong City, Mengyang Town, Sancha River, 4 Aug. 1977, *G. D. Tao 16694* (HITBC036701; HITBC036702); • Xishuangbanna, Mengla County, Menglun Town, Manka Valley, s.d., *P. Z. Zhu 10371* (HITBC036720); • Xishuangbanna, Mengla County, Menglun Town, Modeng, s.d., *G. D. Tao 13701* (HITBC036745); Xishuangbanna, Mengla County, Menglun Town, Baka Small Village, 17 August 2023, *S. Yuan 20230817-1* (IBSC); • Xishuangbanna, Jinghong City, road between Mengkuan and Menglun, 28 October 1978, *G. D. Tao 21756* (HITBC036690); • Dehong, Yingjiang County, trail between Tongbiguan Town and Nabang Town, dense valley forest, 18 June 2014, *L. Wu 4932* (BNU091327; BNU091328); • Dehong, Yingjiang County, Xima Township, Nabangba, moist forest edge, s.d., *G. D. Tao 13148* (HITBC036750); • Dehong, Yingjiang County, Tongbiguan Nature Reserve, 10 October 2011, *S.S. Zhou 11200* (HITBC143065); • ibid., 31 July 2022, *P. Y. Wang, B. Yang, L. Wang & M. L. Chen WPY370* (PE2766094; PE2766095); • ibid., 31 July 2022, *S. S. Zhou, H. B. Ding, X. D. Zeng & L. W. Lin WPY240* (PE2754369); Honghe, Luchun County, 15 km from Daheishan, 15 August 2023, *S. Yuan 20230815-1* (IBSC); **Thailand**. • Phangnga, Kuraburi,22 June 2006, *K. Williams 2067* (E00324136); Ranong, Amphoe Kra Buri, Thungraya Nasak Wildlife Sanctuary, 27 August 2022, *D.J. Middleton, S. Suddee & C. Hemrat 1382* (E00360092; L.0539060; K005313902); Surat Thani. Khao Sok N. P. Trail to Sib Aid Chan waterfall, 15 September 1994, *P. C. Boyce 960* (K005313901); • Rayong, Klang, Kao Chamao National Park, west side, along Chamao Stream, 8 November 1994, *J. F. Maxwell 94-1199* (L2932708; L2932709); • Peninsular. Near Phuket. Thalong, Khao Phra Teo, 19 April 1991, *S. Dawson 19* (K005313904); • Peninsular. Surat Thani Province. Khao Sok National Park, 15 April 1991, *S. Dawson 19* (K005313905); • Surat Thani, Ban Kham Prap, 4 August 1927, *A. Kerr 13161* (K005313911); Trang, Khao Chong, 14 August 1975, *J. F. Maxwell 75-857* (L2932711); • ibid., 11 August 1975, *J. F. Maxwell 75-745* (L2932710); Ranong, 15 km N of Takuapah, evergreen forest, 13 July 1972, *K. Larsen, S. S. Larsen, J. Nielsen & T. Santisuk 30895* (E01525610; P03800621; K005313903); • Nakhon Si Thammarat: Khao Kao, Thung Song, 27 August 1967, *T. Shimizu, N. Fukuoka & A. Nalampoon T8179* (K005313908); • Kanchanaburi, near Neechey (Wangkla), Kwai Noi Basin, stream-side, 16 May 1946, *K. N. Kasin 396* (L2936016; L2936017; K005313899; P03828119); • Trang, Khao Chong forest, 14 September 1933, *D. J. Collins 2385* (K005313900); • Phetburi, Khao Soi Dao, evergreen forest, 29 April 1930, *A. F. G. Kerr 19446* (K005313909); • Trat, Ta Kum, 19 May 1930, *Put 2882* (K005313913); • Phetburi, Khao Daeng, edge of evergreen forest, 19 April 1928, *A. F. G. Kerr 15312* (K005313910); • Chanthaburi, Kao Sabap, 6 July 1927, *Put 907* (K005313906); Yala, Niken Kun Long, edge of evergreen forest, 11 December 1972, *T. Santisuk & B.N. 381* (K005313914); • Chiang Rai, Khun Korn waterfall, 19 June 1999, *PK 181* (BCU); • Phitsanulok, Lom Sak, 13 June 1960, *Smitinand & St.John 6842* (K); Thung Salaeng NP, 20 July 1966, *Larsen et al. 564* (P); **Laos**. • Louang Nantha, Medicine biodiversity reserve, 12 August 2013, *H. Sun, Y. F. Yun, T. Deng Sunhang17955* (KUN1298497); • Vientiane, Vang Vieng, limestone valley under forest, 27 November 2012, *H. Sun, T. Deng, J. W. Zhang, Y. Niu, Q. Wang & Y. S. Chen Sunhang13716* (KUN1258399); • Luang Prabang, vicinity of Sop Keng Village, trail to waterfall SE of village, riverbank beside waterfall, 4 October 2014, *C. Puglisi, M. Rodda & K. Souvannakhoummane LAOS212* (E0095084); **Malaysia**. • Kelantan, Pergau: Sungei Renyok Intake II & III, 08 October 1992, *N. A. Karim & M. Shah NK84* (HUH02489391); • Perak, Tapah: Sungai Chendering, 13 February 1975, *M. Shah, A. Shukor & MS 3413* (L2936018); • Kedah, Baling, 10 May 1938, *M. S. Kiah, Singapore Field No. 35385* (HUH02489392; K005313681); • Selangor; Lemangkok Pass, 22 January 1921, *H.N. Ridley sn*. (K005313684); • Jalan Pokok Asam, Taiping, Perak. 10 April 1968, *Sidek S319* (K005313678).

## Discussion

The present study clarifies the taxonomic status of *Mussaenda wallichii* and two related names, *M. intuspilosa* and *M. lancipetala*. Based on examination of type specimens, protologues, and extensive herbarium materials, we found that the morphological differences previously used to distinguish these taxa fall within the variation range of *M. wallichii*. Therefore, *M. intuspilosa* and *M. lancipetala* are here treated as heterotypic synonyms of *M. wallichii*. The previous recognition of *M. intuspilosa* and *M. lancipetala* as separate species was likely associated with limited examination of available specimens and insufficient understanding of morphological variation within *M. wallichii*. Early taxonomic treatments of *Mussaenda* were often based on a small number of collections, resulting in the overinterpretation of variable morphological characters as species-level differences. The diagnostic characters previously used to distinguish these taxa, including indumentum, number of secondary veins, corolla lobe shape, and calycophyll pubescence, exhibit continuous variation among populations. Although *M. wallichii* was described as having glabrous or nearly glabrous organs, specimens examined in this study showed considerable variation in pubescence density among individuals and developmental stages. Young branches and leaves may possess sparse pubescence, whereas mature organs often become less pubescent or glabrescent. A similar variation in indumentum has been reported in other species of *Mussaenda*, suggesting that this character alone is insufficient for species delimitation (Jayaweera 1963; Chantaranothai 2015). The number of secondary veins was also considered a diagnostic character separating *M. intuspilosa* and *M. lancipetala*. However, our comparison of type specimens and additional collections revealed substantial overlap in this character. The difference reported in the protologue likely resulted from variation in counting minor veins rather than a true diagnostic character. Variations in corolla lobe shape and calycophyll morphology were also observed among examined specimens. Characters such as the degree of acuminate or caudate apices and the density of pubescence on calycophyll veins showed continuous variation rather than discrete differences. These overlapping characteristics further support the conclusion that the three names represent a single morphological entity. The geographical distribution of *M. wallichii* also supports the synonymization proposed here. The species occurs across a broad region extending from northeastern India and Myanmar to Thailand, Laos, southwestern China, and Peninsular Malaysia. The absence of clear geographical discontinuities among populations and the lack of consistent morphological differentiation are consistent with treating the previously recognized taxa as a single widespread species. The Chinese name “狭瓣玉叶金花” is retained for *M. wallichii*, because the name was originally applied to *M. lancipetala*, which is treated here as a heterotypic synonym.

The synonymization presented here contributes to a more accurate understanding of species diversity in *Mussaenda*. Previous estimates of species richness in the genus have been influenced by incomplete knowledge of morphological variation and insufficient examination of type materials. Continued revisionary studies integrating field observations, herbarium research, and molecular evidence will be essential for resolving remaining taxonomic uncertainties within this morphologically diverse genus.

## Supplementary Material

XML Treatment for Mussaenda wallichii

## Additional information

### Artificial Intelligence (AI) use

The authors accept full responsibility for the content of the manuscript, including the disclosure of any use of AI.

No AI tools were used in the preparation of this manuscript.

### Author ORCIDs

T.-T. Duan https://orcid.org/0000-0003-2140-9967

J. Li https://orcid.org/0000-0001-5034-511X

### Data availability

All of the data that support the findings of this study are available in the main text.
